# Validation of the HEART score in Indian population

**DOI:** 10.1186/1865-1380-8-S1-P5

**Published:** 2015-04-22

**Authors:** Balaji Natarajan, Pragya Mallick, Tausif A Thangalvadi, Parivalavan Rajavelu

**Affiliations:** 1 Sundaram Medical Foundation, Chennai, India

## Objectives

Chest pain is a common presentation to the Emergency department (ED) where an acute coronary syndrome needs to be differentiated from other causes of chest pain. In a busy ED, we need an objective method to risk stratify patients quickly, using minimum resources. There are various scoring methods used for this, but none exclusively for ED patients except the HEART score. HEART score stands for History, ECG, Age, Risk factors, and Troponin I where each component is scored from 0 to 2. The goal of this study was to determine a correlation between clinical judgement, HEART score and outcomes in all patients presenting with chest pain to the ED and validate the accuracy of the HEART score in risk stratifying ED chest pain patients.

## Methodology

The study was done at Sundaram Medical Foundation, a 170 bed community hospital in Chennai. It is a prospective study done over a period of 3 months. Inclusion criteria included patients older than 18 years with chest pain. The patients were treated as per the emergency physician’s clinical judgment, and HEART score was calculated for all these patients independent of standard practice of care. However, the treatment was not influenced by the HEART score. Patients were followed up after 1 month for Major Adverse Cardiac Events (MACE). Outcome measures included correlation between the HEART score and occurrence of MACE in the 30 day follow up period.

## Findings

The study enrolled 87 patients, and the Male: Female ratio was 60: 27. 45 patients presented with low HEART score (0-3), 28 with moderate (4-6) and 14 with high score (7-10). In patients with high HEART score 13/14 (93%) had MACE indicating a sensitivity of 93%, in patients with low HEART score 1/44 (2.22%) had MACE indicating a specificity of 98%, in patients with moderate HEART score 12/28 (43%) had MACE.

## Conclusion

HEART score is a simple, accurate and highly reliable tool for stratifying chest pain patients. HEART score closely follows clinical reasoning, is easy to remember, and, therefore, can be used as an adjunct decision tool for ED chest pain patients.

